# Brain region dependent molecular signatures and myelin repair following chronic demyelination

**DOI:** 10.3389/fncel.2023.1169786

**Published:** 2023-04-26

**Authors:** Grace Samtani, Sunja Kim, Danielle Michaud, Andrew E. Hillhouse, Joseph A. Szule, Kranti Konganti, Jianrong Li

**Affiliations:** ^1^Texas A&M Institute for Neuroscience, Texas A&M University, College Station, TX, United States; ^2^Department of Veterinary Integrative Biosciences, Texas A&M University, College Station, TX, United States; ^3^Texas A&M Institute for Genome Sciences and Society, Texas A&M University, College Station, TX, United States; ^4^Department of Veterinary Pathobiology, Texas A&M University, College Station, TX, United States

**Keywords:** chronic demyelination, remyelination, cuprizone, Axonal-glia interaction, multiple sclerosis, motor function, axonal damage, myelin repair

## Abstract

Multiple sclerosis (MS) is the most prevalent demyelinating disease of the central nervous system, characterized by myelin destruction, axonal degeneration, and progressive loss of neurological functions. Remyelination is considered an axonal protection strategy and may enable functional recovery, but the mechanisms of myelin repair, especially after chronic demyelination, remain poorly understood. Here, we used the cuprizone demyelination mouse model to investigate spatiotemporal characteristics of acute and chronic de- and remyelination and motor functional recovery following chronic demyelination. Extensive remyelination occurred after both the acute and chronic insults, but with less robust glial responses and slower myelin recovery in the chronic phase. Axonal damage was found at the ultrastructural level in the chronically demyelinated corpus callosum and in remyelinated axons in the somatosensory cortex. Unexpectedly, we observed the development of functional motor deficits after chronic remyelination. RNA sequencing of isolated brain regions revealed significantly altered transcripts across the corpus callosum, cortex and hippocampus. Pathway analysis identified selective upregulation of extracellular matrix/collagen pathways and synaptic signaling in the chronically de/remyelinating white matter. Our study demonstrates regional differences of intrinsic reparative mechanisms after a chronic demyelinating insult and suggests a potential link between long-term motor function alterations and continued axonal damage during chronic remyelination. Moreover, the transcriptome dataset of three brain regions and over an extended de/remyelination period provides a valuable platform for a better understanding of the mechanisms of myelin repair as well as the identification of potential targets for effective remyelination and neuroprotection for progressive MS.

## Highlights

- Spatiotemporal gene profiles of reparative processes following chronic demyelination.
- Development of motor functional alterations over extended recovery after chronic demyelination.
- Common and unique molecular pathways identified for chronic de/remyelination.

## Introduction

Multiple Sclerosis (MS) is a chronic demyelinating disease of the central nervous system (CNS) with pathological characteristics of inflammation, myelin destruction and axonal transection (Trapp et al., 1998). The loss of myelin sheaths renders the axon vulnerable to damage that disrupts axonal conduction and neural network stability. Axonal deterioration and neuronal atrophy as disease progresses lead to the permanent loss of neurological functions (Mahad et al., 2015; Reich et al., 2018). Accumulating evidence has shown that remyelination of denuded axons restores conduction velocity, improves axonal integrity, and contributes to functional recovery in experimental models (Duncan et al., 2009; Mei et al., 2016; Franklin and Ffrench-Constant, 2017). As such, enhancing remyelination in MS has been considered an important strategy to protect axons and restore conduction. However, while endogenous remyelination can occur in the early stages of MS, this process is often incomplete and ultimately fails at later stages (Franklin and Ffrench-Constant, 2017). It is thus critical to understand cellular and molecular pathways involved in myelin repair, particularly in the context of chronic demyelination, and to identify factors that promote effective myelin repair prior to the permanent loss of neurological functions.

Remyelination, a regenerative process involving the production of new oligodendrocytes (OLs) from oligodendrocyte progenitor cells (OPCs) and the formation of new myelin sheaths around the demyelinated axon, has been investigated in various experimental models of CNS demyelination, including the OL toxin cuprizone model (Franklin and Ffrench-Constant, 2017). Cuprizone, a copper chelating agent that is administered as rodent dietary supplement, induces astroglial/microglial activation, OL apoptosis and demyelination in multiple brain areas without blood-brain barrier disruption and the involvement of an autoimmune process (Matsushima and Morell, 2001; Kipp et al., 2009; Steelman et al., 2012; Gudi et al., 2014). Remyelination takes place spontaneously following the withdrawal of cuprizone from the diet, allowing the elucidation of the fundamentals of CNS regenerative biology after demyelination. While endogenous remyelination following acute demyelination has been investigated extensively, the regenerative processes following chronically active demyelination are much less studied. Prolonged exposure to cuprizone (e.g., 12 weeks) leads to chronic demyelination in cerebral gray matter (Skripuletz et al., 2008) and white matter, where the remyelination process is delayed and inefficient when compared to that after acute injury (Armstrong et al., 2006). Delineating CNS regenerative processes following the progression of acute to chronic demyelination shall contribute to our understanding of disease progression and potential development of therapeutic strategies for progressive MS. However, the molecular determinant of myelin repair of chronically demyelinating lesions remains poorly understood. Moreover, few studies have examined behavioral functional outcomes after chronic cuprizone-induced demyelination and during the recovery phase of myelin repair (Sen et al., 2019; Lubrich et al., 2022).

In this study, we systematically examined myelin repair processes following acute and chronic cuprizone exposure focusing on spatiotemporal cellular and molecular analyses in the context of chronic de/remyelination and differences between acute versus chronic phases. We characterized gene expression changes in both cerebral white and gray matter, namely the corpus callosum, cortex, and hippocampus through an extended course of chronic de/remyelination, and identified differential repair processes and brain region heterogeneity in remyelination after chronic cuprizone intoxication. Furthermore, using transgenic myelin reporter mice where membrane-tethered EGFP is selectively expressed in mature OLs (Deng et al., 2014), we visualized myelin sheaths during the remyelination phase of chronic demyelination and uncovered significant focal axonal damage despite of the return of myelin. Ultrastructural analysis of the callosum white matter revealed axonal pathology in chronic de/remyelination and confirmed significant extent of remyelination of chronically demyelinated lesions after extended recovery. Moreover, we examined motor functions of mice following chronic demyelination and during an extended recovery period up to 12 weeks after cuprizone withdrawal, and unexpectedly found new functional deficits developed in parallel with chronic remyelination. Analysis of differentially expressed genes in chronically de/remyelinated white matter suggests activation of specific molecular pathways. These findings provide new insights into endogenous CNS regenerative mechanisms following chronic demyelination. The gene profiles of three brain regions over an extended de/remyelination period provide a foundation for further investigation into myelin repair and axonal protection and towards the development of therapeutic strategies for progressive MS.

## Materials and methods

### Animals

CNP1-mEGFP mice were generated and characterized previously (Deng et al., 2014). C57BL/6J mice were purchased from the Jackson Laboratory (Bar Harbor, ME, USA) and allowed acclimation for at least 1–2 weeks before experimentation. All mice were maintained under standard housing conditions in a temperature-controlled room on a 12-h light/dark cycle with access to standard food and water *ad libitum.* All experiments were performed in accordance with the guidelines of the National Institutes of Health and were approved by the Institutional Animal Care and Use Committee at the Texas A&M University.

### Cuprizone induction of acute and chronic de/remyelination

Cuprizone-induced demyelination was achieved as described previously (Kim et al., 2018) by feeding adult mice (8–10 weeks) cuprizone (CPZ, bis-cyclohexane oxal-dihydraxone) milled into standard chow at the concentration of 0.2% (Harlan-Teklad, TD.140800) for 3–5 weeks (wk) for acute or 12 weeks for chronic demyelination. Mice were weighed every other day to monitor the characteristic weight loss during the first 10 days (Steelman et al., 2012; Kim et al., 2018). Fresh cuprizone chow was provided every other day. At the end of cuprizone feeding, mice were returned to their normal diet for a recovery period of 0–12 weeks as specified. Both male and female mice were used and were randomly and evenly assigned to experimental groups with various recovery periods. Recovery periods are denoted with “ + “ followed by the number of weeks returned to the normal diet (i.e., 5 + 1 week). Age-matched naïve control mice were kept on the standard diet throughout the duration of the cuprizone experiment. C57BL/6J mice were subjected to behavioral testing at specified timepoints and/or sacrificed for immunohistochemical analysis, RNA extraction for deep sequencing, or electron microscopy. CNP1-mEGFP mice were used for immunohistochemical analysis and direct visualization of myelin sheaths and mature oligodendrocytes. BioRender was used to generate experimental schemes.

### Mouse brain sample preparation for RNA extraction, sequencing, and analysis

Mice were anesthetized followed by transcardial perfusion with 20 ml of sterile PBS (Kim et al., 2012; Steelman et al., 2012). The brain was removed, and the corpus callosum was quickly but meticulously dissected using a sterile razor blade. For chronic de/remyelination studies, the cortex and hippocampus were also microdissected. Each piece of tissue was flash frozen in liquid nitrogen and stored at −80°C until use. RNA was extracted with Tri Reagent (T9424, Sigma Aldrich) from the corpus callosum of cuprizone-intoxicated mice at 3, 5, and 5 + 1 week (5 week cuprizone followed by 1 week of recovery) and from the corpus callosum, cortex, and hippocampus of normal and cuprizone-intoxicated mice at 12, 12 + 4, 12 + 8, and 12 + 12 week.

The mRNA libraries were prepared using the TruSeqRNA Sample Prep Kit (Illumina). For the acute and chronic CPZ cortex samples, forty base-pair reads were generated for each sample on an Illumina HiSeq 2500 instrument at the Whitehead Institute Genome Technology core facility. Chronic CPZ corpus callosum and hippocampus samples were sequenced on a one hundred fifty base pair, paired-end sequencing run on an Illumina NovaSeq 6000 at the Texas A&M Institute for Genome Sciences and Society. Between 22 and 35 million reads were generated per sample. The RNA-Seq data have been deposited in the National Center for Biotechnology Information (NCBI) Gene Expression Omnibus (GEO) and are accessible through GEO Series accession number GSE212147. Reads were mapped to the mouse genome (GRCm38/mm10) using TopHat version 2.0.13 with default settings. Normalized fold-changes of all samples were achieved using DESeq2, and were used to determine differential gene expression between samples. Genes with differential expression (Log2 FoldChange (FC) > 1, *p* value < 0.05, and basemean > 10) were fed into Ingenuity Pathway Analysis (IPA, Qiagen) for canonical pathway enrichment and comparison analyses. Unbiased clustering and heatmaps were generated with normalize datasets (log2 transformation) processed with the pheatmap R package (v1.0.12). Detailed cut off criteria for each Heatmap are described in the figure legends.

To compare gene expression changes in brain regions and during the course of de- and re-myelination, DeSeq2 data were compared using a venn diagram.^1^ Gene Set Enrichment Analysis (GSEA) was performed using the Broad Institute GSEA software (Subramanian et al., 2005). Genes were ranked based on log2 FoldChange differences and q-value between two populations (Clark et al., 2021). The significance of gene sets (Molecular Signature Database C5, version 7.1) was estimated based on the normalized enrichment score (NES). For each gene set, 1,000 permutations were used to calculate the probability of the gene with the given NES.

### Immunohistochemistry

Mouse brains were collected after transcardial perfusion with 20 ml of sterile PBS, post-fixed in 4% paraformaldehyde (PFA) overnight at 4°C, and cryoprotected in 30% sucrose solution until sunk. The tissues were sectioned at 10 or 30 μm thickness with Leica CM1950 cryostat into coronal (whole brain) or sagittal (one hemisphere) sections and mounted onto super-frost plus microscope slides (Fisher Scientific, #12-550-15). Tissue slides were dried at 37°C for 30–60 min, rehydrated in PBS, and permeabilized with 0.3% Triton X-100 in PBS (PBS-T) for 15 min. Brain sections were blocked with 0.1% PBS-T containing 5% goat serum (or donkey serum when goat primary antibody was used) for 1 h at room temperature, and incubated with primary antibodies at 4°C overnight. The following primary antibodies were used: goat anti-Iba1 (1:500, Abcam ab5076), rat anti-GFAP (1:500, Invitrogen #13-0300), rabbit anti-NeuN (1:50 Cell Signaling #24307), mouse anti-APP (1:200, Cell Signaling #2450), mouse anti-SMI-32 (1:200, BioLegend, #801701), mouse anti-synaptophysin (1:200, Sigma S5768), guinea pig anti-vGluT1 (1:5000, Millipore AB5905) or rat anti-PDGFRa (1:100, BD Pharmingen #558774). Following incubation with primary antibody, slides were washed in PBS-T and further incubated for 1 hr at room temperature with secondary antibodies conjugated with AlexaFluor 488, 555, 594, or 647 (1:1,000, Invitrogen). After washes, nuclei were stained with 1 μg/ml Hoechst 33285 and the tissue sections were mounted with ProLong Diamond Antifade Mountant (EGFP tissues) (ThermoFisher P36961) or Fluoromount-G (SouthernBiotech, 100241-874) and air-dried overnight in the dark prior to imaging.

### Immunofluorescence microscopy and image analysis

Epifluorescence images were captured with a fluorescence microscope equipped with an Olympus DP70 digital camera (model IX71; Olympus, Tokyo, Japan) or an Axio Imager M2 microscope (Carl Zeiss Inc., USA). Confocal images were taken with a Zeiss LSM 780 Confocal Microscope. The NIH Image J program was used for image analysis including integrated density measurement and cell counting as detailed previously (Kim et al., 2018). An average of three sections per animal in the area of the medial corpus callosum and somatosensory cortex (layers 1-6) were used for quantitative analysis. Myelin was assessed as mEGFP^+^ or PLP^+^ signal in the region of interest (ROI) and analyzed for density after images were transformed to binary images using ImageJ. Myelin density was quantified as percentage of EGFP- or PLP-positive area of the total area of the ROI and was tabulated relative to normal myelin density of naive mice. Microglial cell number was counted as nucleated Iba1^+^ cells using the 3D object counter in ImageJ. The level of astrocyte activation was quantified via integrated GFAP^+^ density measurement of binary images as detailed previously (Steelman et al., 2012). Rolling ball radius was used for background removal prior to quantification. Axonal damage was evaluated by relative integrated density measurements of APP^+^ signal.

### Behavioral testing

On the day of behavior tests, mice were habituated for at least 30 minutes in the testing room. The following tests were conducted for the chronic C57BL/6J mouse groups: prior to cuprizone intoxication, at 12 weeks of cuprizone feeding, and at all recovery timepoints (12 + 4 week, 12 + 8 week, and 12 + 12 week). All behavior tests were performed during morning hours and chambers and objects were cleaned thoroughly with 70% ethanol between animals.

*Open Field:* This test was used to evaluate general mouse locomotor activity, anxiety and exploration habits (Franco-Pons et al., 2007). Mice were placed in the center of the plexiglass arena (26 cm × 26 cm) of the Truscan Activity System (Coulbourn Instruments, Holliston, MA, USA). Horizontal and vertical movements were recorded by the Truscan 2.0 software at 2-min intervals for 30 min. Analyzed parameters include total number of moves, total move time, total rest time, total distance traveled, velocity, margin distance, margin time, center distance, center time, center entries and jumps, and vertical plane moves, distance, time and entries.

*DigiGait:* This test was used to assess gait alterations (Amende et al., 2005). Briefly, mice were placed on the DigiGait (Mouse Specifics, Inc.) treadmill set at a 0° incline/decline and run at a speed of 24 cm/s for about 30 s, or until five consecutive steps were captured on the DigiGait Imager. Immediately prior to running, mice acclimated to a lower speed (15–20 cm/s) for a few seconds. Steps were analyzed with the DigiGait Analyzer, which required definition of left and right forepaws and hindpaws. All metrics were exported and analyzed individually for significance. Selected methods include the stride frequency (number of times per second a paw makes a stride), stride length (distance between successive strides of the same paw), stance width (perpendicular distance between each set of paws at peak stance), paw angle (degree of external rotation), and the ataxia coefficient (step-to-step variability between all four paws), with definitions based on the manufacturer’s manual.

### Transmission electron microscopy

A subset of C57BL/6J mice at 12 and 12 + 12 week (*n* = 4–5/timepoint) was transcardially perfused with PBS and fixed with 2% paraformaldehyde and 2% glutaraldehyde in PBS (Electron Microscopy Sciences, Hatfield, PA) for 10 min. The brains were removed and kept in the fixative solution overnight at 4°C. The brains were then segmented at the medial sagittal plane and trimmed into 1–2 mm thickness sagittal sections and kept in 0.1 M sodium cacodylate buffer on ice. After washing, the tissues were post-fixed in 1% osmium tetroxide in 0.1 M sodium cacodylate buffer, dehydrated in an ascending ethanol series and embedded in either Embed 812 or Araldite epoxy resin (Electron Microscopy Sciences, Hatfield, PA, USA). Tissues were cut into semi-thin sections (400 nm thickness) and stained with Toluidine Blue for visualization, and ROIs were determined. Ultra-thin sections (100 nm thickness) containing the medial mid-caudal corpus callosum were cut with a Leica EM UC6 ultramicrotome and diamond knife (DiATOME; Hatfield, PA, USA), post-stained in 2% aqueous uranyl acetate followed by lead citrate and examined with an FEI Morgagni 268 transmission electron microscope. Digital images were acquired with a MegaViewIII camera operated with iTEM software (Olympus Soft Imaging Systems, Germany).

### Statistical analyses

All analysis was performed using GraphPad Prism Version 6.01 (GraphPad Software, San Diego, CA, USA). The D’Agostino & Pearson normalcy test was run between two columns. The parametric, unpaired student’s t-test was run to compare significance between two groups. One-way analysis of variance (ANOVA) followed by Tukey’s *post-hoc* test was used for a significance comparison when comparing more than two groups. A confidence interval of 95% was set for each statistical measure with significance at *p* < 0.05. Results were represented as mean ± SEM.

## Results

### Spontaneous remyelination occurs following demyelination induced by acute and chronic cuprizone exposure

**FIGURE 1:**
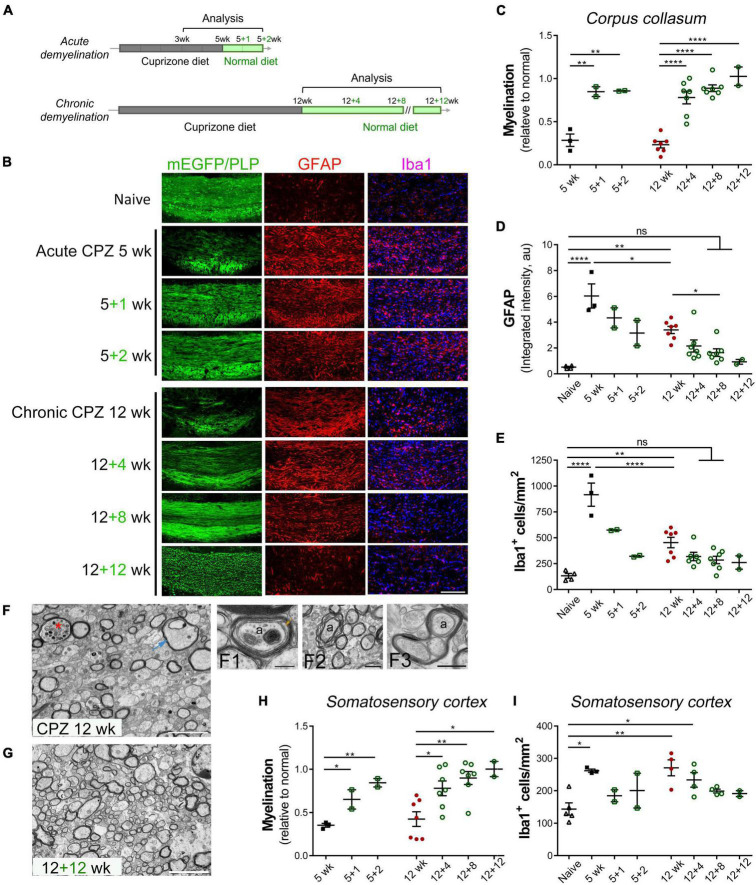
Spontaneous remyelination follows acute and chronic demyelination in the cuprizone mouse model. **(A)** Schematic overview of acute and chronic cuprizone treatment (demyelination) and recovery (remyelination). Adult mice were fed cuprizone diet (CPZ) for 5–12 weeks, followed by recovery with normal diet for up to 12 weeks. Mice were sacrificed at indicated time points for analysis. **(B)** Representative images of the medial corpus callosum directly visualized for CNP-driven mEGFP expression and immunostained for GFAP, Iba1, or PLP in the case of 12 + 12 weeks. Scale bar, 200 μm. **(C)** Quantitative analysis of myelination in the medial corpus callosum of mice treated as specified. The level of myelination was assessed based on the percentage of mEGFP^+^ or PLP^+^ area in the medial corpus callosum in comparison to that of naïve mice. **(D,E)** Quantitative analyses of astrocyte activation based on GFAP integrated density in arbitrary units (au) and of microgliosis based on Iba1^+^ cell counting. Data are mean ± SEM, and data points represent individual mice. *p < 0.05; **p < 0.01; ****p < 0.0001; ns, not significant. One-way ANOVA with Tukey’s *post-hoc* correction. **(F,G)** Representative transmission electron microscopy images of the medial corpus callosum region of mice undergone chronic de/remyelination (12 weeks CPZ, 12 + 12 weeks, respectively). Data represent at least three mice per group. Asterisk denotes a myelinated axon with luminal accumulation of organelles, and arrow indicates a swelling axon with vacuolation. Also shown at higher magnification are a representative axon (a) undergoing active remyelination with a proceeding inner tongue (arrow) and two complete wraps of compacted major dense lines (F1), myelinic splitting (F2), and apparent aberrant enlargement of oligodendrocyte loops (F3). Ax, axon. Scale bars, 5 μm **(F,G)**, 200 nm (F1), 1 μm (F2), 500 nm (F3). **(H,I)** Quantitative analysis of de/remyelination based on mEGFP signal and Iba1^+^ cells density in the somatosensory cortex following acute and chronic demyelination. Data represent mean ± SEM, and data points represent individual mice. *p < 0.05; **p < 0.01. One-way ANOVA with Tukey’s *post hoc* correction.

To investigate remyelination processes following chronic demyelination, we employed the cuprizone animal model that is independent of autoimmunity and produces demyelination reminiscent to certain characteristics of pattern III MS lesions (Matsushima and Morell, 2001; Steelman et al., 2012). Cuprizone, a copper chelator administered as a dietary supplement to adult mice, is thought to selectively induce metabolic stress in mature OLs, leading to oligodendropathy and astroglial and microglial activation followed by demyelination in distinct brain regions, predominantly the corpus callosum. Spontaneous repair of demyelinated lesions ensues rapidly upon cuprizone withdrawal but slows down with lesion chronicity. To determine endogenous remyelination capacities, we fed adult *CNP1-*mEGFP and C57BL/6J mice with rodent chow containing 0.2% cuprizone for 5 weeks to achieve maximum acute demyelination of the corpus callosum or for 12 weeks to achieve chronic demyelination. Mice were returned to their normal diet for 1–2 weeks after 5 week cuprizone exposure (5 + 1, 5 + 2 week, acute de/remyelination paradigm) or for 4–12 week after 12 weeks of chronic cuprizone exposure (12 + 4, 12 + 8, 12 + 12 weeks, chronic de/remyelination paradigm) (Figure 1A).

We used transgenic CNP1-mEGFP mice, which express membrane-anchored enhanced green fluorescence protein in mature oligodendrocytes, to directly visualize myelin sheaths and membrane architecture (Deng et al., 2014). For C57BL/6 mice, immunohistochemistry for myelin proteolipid protein (PLP) was used to access the level of myelination. Consistent with prior studies (Matsushima and Morell, 2001; Gudi et al., 2014), cuprizone intoxication for either 5 or 12 weeks resulted in significant demyelination of the medial corpus callosum in comparison to naïve controls on the normal diet (Figures 1B, C). The loss of myelin was associated with significant activation of astrocytes and microglia/macrophages as evaluated by GFAP upregulation and Iba1^+^ cell counting, respectively (Figures 1B, D, E). Chronically demyelinated lesions had markedly lower levels of gliosis in comparison to acutely demyelinated lesions (Figures 1B–E, 12 weeks vs. 5 weeks). Significant remyelination occurred following both acute and chronic demyelination although with different kinetics as demyelination associated loss of mEGFP or PLP immunosignal was largely restored to a level close to that of controls after 1–2 weeks recovery following acute cuprizone treatment or after 4–8 weeks recovery following chronic cuprizone exposure (Figure 1C). Concomitant with myelin regeneration was the decrease in gliosis particularly in the acute de/remyelination paradigm (Figures 1D, E).

As the mEGFP or PLP immunoreactivity was largely recovered a few weeks after acute and chronic demyelination, we next used transmission electron microscopy (TEM) to confirm remyelination and examine axon/myelin at the ultrastructural level. We previously reported that the caudal medial corpus callosum underwent extensive demyelination upon 5 weeks of cuprizone exposure with pronounced myelin debris and lipid droplets often found in microglial cells and that by just 2 weeks of recovery, substantial callosal axons underwent remyelination (Steelman et al., 2012). In contrast to acute cuprizone treatment where there was minimum endogenous remyelination at the 5 weeks timepoint, 12 weeks of continued cuprizone treatment triggered myelin regenerative responses concomitant with ongoing demyelination as evidenced by multiple axons were thinly remyelinated (Figures 1F, F1). Myelin debris and lipid droplets were rarely found in the chronic demyelination paradigm. However, we observed, in an area-dependent manner, ultrastructural axon/myelin aberrations including axonal swelling and luminal organelle accumulation, myelinic splitting, and enlargement of OL loops or the inner tongue (Figures 1F, F2, F3). After 12 weeks of cuprizone-free recovery, significantly more axons were remyelinated (Figure 1G). Together, these results demonstrate that the adult brain possesses innate remyelination capabilities even following a chronic demyelinating insult.

As cuprizone induced demyelination exhibits spatial-temporal heterogeneity in several brain regions including cerebral gray matter (Skripuletz et al., 2008; Gudi et al., 2009) and as cortical lesions represent a key manifestation of MS and contribute to chronic MS disabilities, we next examined cortical gray matter following cuprizone exposure with a focus on the somatosensory cortex, which has been implicated in impaired somatosensory gating and walking performance in MS (Arpin et al., 2017). Compared to controls, acute and chronic cuprizone intoxication resulted in significant loss of endogenous mEGFP or PLP immunosignal across layers 1–6 of somatosensory cortices (Figure 1H), and patchy appearance of compact myelin in the cortical layer 3 was more evident when visualized with mEGFP (Supplementary Figure 1). Similar to remyelination in the corpus callosum during the recovery phase, cortical remyelination also occurred progressively over time (Figure 1H and Supplementary Figure 1). However, cortical microgliosis induced by acute demyelination was much less robust than that of the corpus callosum (Figures 1E, I). Interestingly, unlike the corpus callosum, the number of microglial cells in the somatosensory cortices was increased to a similar extent between acute and chronic demyelination (Figure 1I), suggesting regional specificity of microglial responses. Taken together, our results are consistent with previous findings (Matsushima and Morell, 2001; Gudi et al., 2014) and suggest that myelin regenerative processes differ in different brain regions and that endogenous remyelination, despite at a slower recovery rate, can be extensive even after chronic exposure to a demyelinating insult.

### Cortical axonal damage persists after chronic demyelination and remyelination and the development of new functional deficits in bilateral sensorimotor coordination during recovery

**FIGURE 2:**
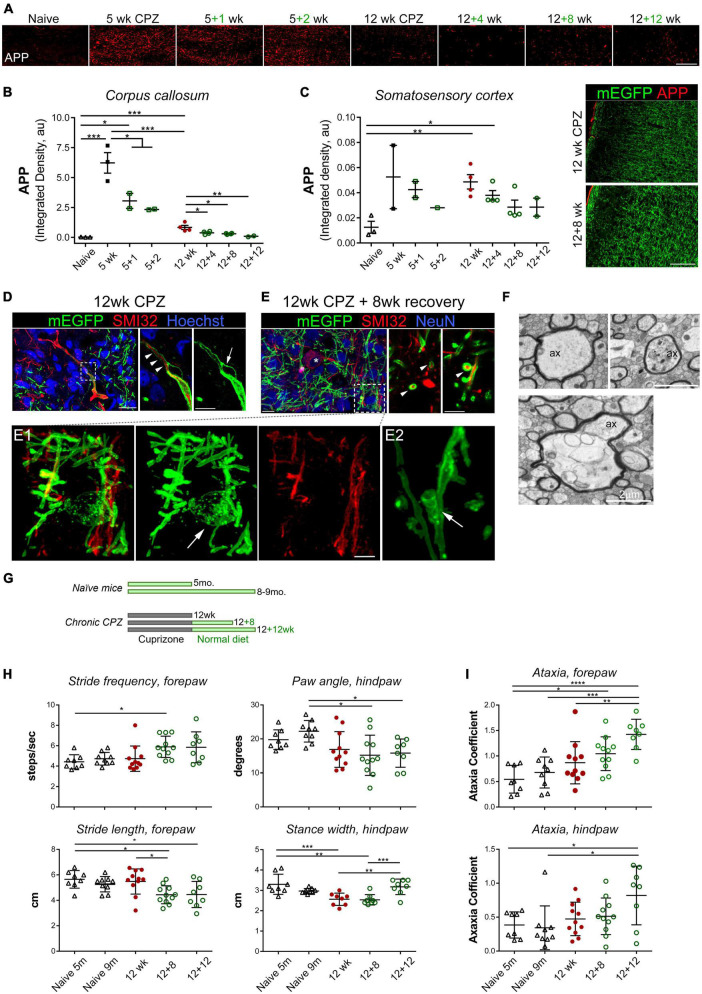
Chronic demyelination is associated with axonal damage and the development of bilateral sensorimotor deficits during extended recovery. **(A)** Representative APP immunostaining images of the medial corpus callosum of control and mice treated with cuprizone for 5 and 12 weeks followed by 1–12 weeks of recovery. **(B,C)** Quantification of axonal damage based on integrated density of APP immunoreactive signal in the medial corpus callosum **(B)** and somatosensory cortex **(C)**. Data represent mean ± SEM of individual mice. *p < 0.05; **p < 0.01; ***p < 0.001. One-way ANOVA with Tukey’s *post-hoc* correction. For 5 weeks versus 12 weeks, unpaired Student’s *t*-test. **(D)** Maximum projection of confocal images of immunostained cortex of CNP-mEGFP mice treated with cuprizone for 12 weeks, showing a representative SMI32^+^ axon with mEGFP^+^ myelin sheaths (left, z-stack depth: 12 μm). Single z-stack plane of the boxed area is zoomed-in on the right. Arrowheads indicate a SMI32^+^ axon remaining myelinated, and arrow denotes a bubble-like splitting of myelin sheaths of the SMI32^+^ axon. Scale bars, 20 μm (left), 10 μm (right). **(E)** Confocal, images of SMI32 and NeuN immunostained cortex sections from CNP-mEGFP mice treated with cuprizone for 12 weeks followed by 8 weeks of recovery (z-stack depth, 9.5 μm). Asterisk indicates a SMI32^+^ NeuN^+^ double positive neuron. Right, confocal photomicrographs at single z-stack plane of representative cross-section view of myelinated SMI32^+^ axons (arrowheads). A myelinating mEGFP^+^ oligodendrocyte highlighted in the boxed area is shown in panel (E1) at a higher magnification. Panel (E2), an example of mEGFP^+^ oligodendroglial process spirally enwrapping axon. Scale bars, 10 μm (left), 5 μm (right), 2 μm (E1). **(F)** TEM photomicrographs of representative axonal damage and aberrations of the axon-oligodendrocyte unit observed following 12 weeks of cuprizone treatment. Ax, axon. Scale bars, 2 μm. **(G–I)** Experiment scheme and DigiGait behavioral analysis of mice after chronic de/remyelination and their age-matched controls. Five-month-old naïve mice corresponded to mice that were intoxicated with cuprizone for 12 weeks and 9-month-old naïve mice corresponded to mice undergone 12 + 12 weeks demyelination and recovery. Data are mean ± SD of averaged stride frequency and stride length of forepaws of individual mice and absolute paw angles and averaged stance width of hind paws of each animal **(H)**, and ataxia coefficient of forepaws and hind paws of individual mice **(I)**. Data points represent individual animals (*n* = 8–11 mice per time point). *p < 0.05; **p < 0.01; ***p < 0.001; ****p < 0.0001. One-way ANOVA with Tukey’s *post-hoc* correction.

Electron microscopic analysis revealed frequent ultrastructural swelling of axons following chronic demyelination (Figures 1F, 2F). To evaluate axonal damage, we examined accumulation of beta-amyloid precursor protein (APP), a marker indicative of impaired axonal transport and axonal injury, across acute and chronic de/remyelination timepoints (Figures 2A–C). Integrated APP density was most upregulated in the caudal corpus callosum after acute demyelination, in agreement with the extent of demyelination and gliosis, and remained elevated after 2 weeks of recovery in comparison to controls that had minimum APP immunoreactivity (Figures 2A, B). Chronic demyelination also led to significantly higher levels of APP expression, which gradually lessened over the recovery period (Figures 2A, B). Although chronic demyelination did result in axonal damage, it was much less severe than that of acute demyelination (Figure 2B). APP^+^ spheroids in somatosensory cortices were overtly much less frequent than those observed in the corpus callosum, but consistently greater than the controls (Figure 2C), suggesting certain levels of cortical axonal damage in chronic demyelination.

We next examined cortical axons in more details with direct visualization of OL/myelin sheaths enabled by membrane-anchored mEGFP and immunostaining with the monoclonal antibody SMI-32, which recognizes non-phosphorylated neurofilaments that are particularly enriched in axonal ovoids at the site of axonal transections in MS (Trapp et al., 1998), and as such is often used as a surrogate marker for axonal damage. We found multiple SMI-32–positive axon segments and axonal swellings after 12 weeks of cuprizone treatment, and that the majority of SMI-32^+^ axons at this time point were unmyelinated (Figure 2D). Local degeneration of mEGFP^+^ myelin sheaths and bulging were observed along some SMI-32^+^ axonal swellings, especially the large swelling ones (Figure 2D and Supplementary video 1). Following 8 weeks of recovery when remyelination was active and ongoing, we found, rather unexpectedly, frequent occurrence of myelinated SMI-32^+^ axon segments (Figure 2E and Supplementary video 2). Myelinating oligodendrocytes at this time point, readily identifiable through their membrane-tethered mEGFP signal, exhibited fine membranous architectures, membrane vesicles along fine processes, and spiral wrapping of SMI-32^+^ axons (Figures 2E1, E2). These results suggest that damaged axons, but not large SMI-32^+^ ovoids, can be remyelinated even after a chronically demyelinating insult. Our data also demonstrate the advantage of using the CNP1-mEGFP mice for direct visualization of myelin sheaths and oligodendrocyte membranes *in situ*.

The pronounced, persistent cellular deficits following acute and chronic cuprizone treatment led us to investigate whether they were accompanied by functional deficits *in vivo*. Most studies assessing behavioral deficits after cuprizone exposure have focused on the acute demyelination paradigm and motor function deficits and/or psychiatric effects such as social and anxiogenic behaviors (Sen et al., 2019; Lubrich et al., 2022). Using a complex wheel running test, Hibbits et al. (2009) showed impaired bilateral sensorimotor coordination in cuprizone-treated mice and function recovery upon remyelination following acute demyelination. We thus decided to evaluate the motor function of our cuprizone mice following chronic demyelination and extended recovery with the DigiGait motorized treadmill analysis system, which detects subtle gait alterations, as well as with the open field testing (Figures 2G–I and Supplementary Figure 2). Naïve mice on the normal diet at age of 5 mo. and 9 mo. were used as age-matched controls, corresponding to the age of mice at the end of 12 weeks of cuprizone treatment and at the end of 12 weeks of cuprizone plus 12 weeks of recovery, respectively.

DigiGait analyses, measured between the individual forepaws and hindpaws, include various walking and coordination metrics, such as the distance between consecutive steps of the same paw (stride length), the perpendicular distance between either set of paws (stance width), the amount of times a step is taken (stride frequency), the degree of paw rotation (absolute paw angle), coordination of stepping frequency between the forepaws and hindpaws (gait symmetry), and the variability between forepaw and hindpaw steps (ataxia coefficient). Unexpectedly, of all the parameters measured by the DigiGait system, we did not find significant differences between the control mice and the 12 weeks chronic demyelinated group. The only significant demyelination-dependent alteration between the control and 12 weeks cuprizone mice was decreased stance width between the hindpaws (Figure 2H), which gradually reversed back to the normal upon 8–12 weeks of recovery (Figure 2H). Surprisingly, we found recovery-dependent alterations such as the stride length of forepaws was significantly decreased in the 12 + 8 weeks recovery group when compared to naïve or 12 weeks CPZ group while the stride frequency was concurrently and moderately increased (Figure 2H). Most strikingly were progressive and significant increases of the ataxia coefficient, an index of variability between steps, as remyelination proceeded over the extended recovery period (Figure 2I). Ataxia in the forepaws was more pronounced than that of the hindpaws (Figure 2I). Together, the data from these functional analyses suggest that while remyelination rescues certain sensorimotor functional deficits following chronic demyelination, it is also associated with the development of new, likely adaptive, alterations in bilateral sensorimotor coordination that are not seen in the chronically demyelinated group.

### Unbiased tissue RNA-sequencing reveals shared and distinct remyelination gene profiles of the corpus callosum following acute and chronic demyelination

**FIGURE 3:**
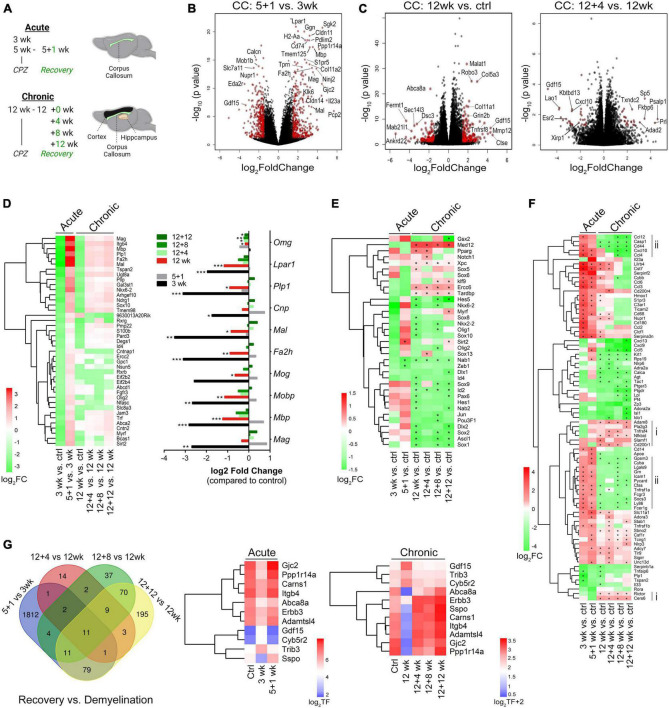
Transcriptome landscapes of the corpus callosum following acute and chronic demyelination and temporal changes during chronic remyelination. **(A)** Schematic of experiments outlining timelines for acute and chronic cuprizone treatment and recovery and brain regions used for RNA-seq. **(B,C)** Volcano plots depicting differential gene expression (DEGs) and *p*-values between acute remyelination and demyelination samples [5 + 1 vs. 3 weeks, **(B)**], and chronic demyelination vs. control samples [12 weeks vs. control, (left **C**)] and early chronic remyelination vs. demyelination samples [12 + 4 vs. 12 weeks, (right **C**)]. Colored symbols indicate significantly differentially expressed genes with Log2 FC (fold changes) > 1.5 and *p* < 0.05. (**D**, Left) Heatmap of significant OL-enriched DEGs (*q* < 0.05, total 41 genes) at various time points after acute and chronic demyelination. Right, relative fold changes of 10 myelin genes over controls. *q < 0.05, **q < 0.01, ***q < 0.001. **(E)** Heatmap of transcription factors/regulators involved in OL lineage cell development and differentiation that were significantly modulated during de/remyelination. Asterisks (*) indicate statistically significant gene changes compared to the controls (*q* < 0.05). **(F)** DEGs that function in the regulation of immune processes and inflammatory responses were analyzed (GO:0006954). Genes were ranked by log2FC and -log10(*p*-value), with cut-off values of (+6, –6) and FC < 1 across all 6 sample groups. The Heatmap of the top-ranked 81 DEGs shows relative differences (log2 FC) of specific samples vs. controls. Asterisks (*) indicate statistically significant gene changes compared to the controls (*q* < 0.05). Vertical lines indicate gene clusters uniquely up- (i) and down-regulated (ii) in chronic de/remyelination samples. **(G)** Venn diagram showing relative overlapping DEGs (*p* < 0.01) among acute and chronic remyelination over demyelination groups. Gene expression levels of the 11 genes common to all comparison groups are visualized as log2 transformed counts.

Our immunohistochemical, TEM, and behavioral results indicate that myelin regeneration after acute and chronic demyelination may be differentially regulated and is insufficient to rescue axonal damage during chronic demyelination. Moreover, our data showed that new motor coordination deficits developed in the context of chronic demyelination, which may result from aberrant or adaptive myelination during the recovery process. To better understand the mechanisms of myelin repair and identify key molecular pathways, we performed a series of bulk RNA-sequencing (RNA-seq) of microdissected white matter and gray matter brain regions from the acute and chronic demyelination paradigms (Figure 3A). Specifically, we isolated the corpus callosum in the acute paradigm, and the corpus callosum, cortex, and hippocampus in the chronic paradigm (Figure 3A). This strategy allows temporal analysis and comparison of gene profiles of the corpus callosum during acute versus chronic remyelination as well as an investigation into brain region-dependent transcriptomic changes during chronic de/remyelination. To study acute demyelination, we chose 3 weeks of cuprizone exposure when myelin-related mRNAs are downregulated whereas genes associated with gliosis are already upregulated (Kim et al., 2018), and one week recovery following 5 weeks of cuprizone intoxication (5 + 1 weeks) as an acute remyelination time-point when robust oligodendrogenesis occurs (Matsushima and Morell, 2001; Steelman et al., 2012).

To establish transcriptional signatures of the corpus callosum during remyelination, we evaluated differentially expressed genes (DEGs) during both acute and chronic phases (Supplementary Tables 1, 2, respectively). Volcano plots of the DEGs between 5 + 1 weeks vs. 3 weeks samples revealed highly significant upregulation of genes associated with myelinating oligodendrocytes, including *Mag, Mbp*, tight-junction claudins (*Cldn14, Cldn11*), gap-junction connexin-47 (*Gjc2*) and connexin-32 (*Gjb1*), Pdlim2, lysophosphatidic acid receptor 1 (Lpar1), fatty acid hydroxylase (Fa2h), and gelsolin (Gsn) (Figure 3B, and Supplementary Table 1). The dataset also revealed other highly upregulated genes during active remyelination including *Ninj2* (Ninjurin 2) (Noroozi et al., 2019; Sun et al., 2022) and tropinins *Tnni1* and *Tnnt1*, suggesting their potential unrecognized roles in myelin repair. When the dataset was cross-compared with cell type-enriched genes (Zhang et al., 2014), it became evident that the most robust transcriptional changes during acute remyelination (5 + 1 wk) versus demyelination (3 wk) were those of oligodendrocytes (Supplementary Figures 3A–C and Supplementary Table 1). We further identified a number of microglia-enriched genes that were differentially expressed during remyelination, of which the most pronounced were those associated with innate immune responses (e.g., *Spp1*, *Cd180)*, scavenging/phagocytosis (e.g., *Msr1, Fcrls, CD33, Mpeg1, Tcirg1*) and antigen recognition/processing (e.g., *H2-Aa, CD74, H2-Eb1, Dok3, Lag3, Slc11a1, Tmem52*) (Supplementary Figure 3D, F–H). Furthermore, remyelination after acute demyelination was associated with increased metabolism of amino acids, demonstrated by the large upregulation of astrocyte-enriched *Gpt* (glutamate pyruvate transaminase 1) and *slc7a10* (Supplementary Figure 3E), and the downregulation of phagocytic astrocytes (e.g., *Gulp1* and *Abca1)* (Supplementary Figure 3E) (Morizawa et al., 2017). Moreover, astrocyte-enriched genes known to modulate synaptic function and extracellular matrix interactions such as metabotropic glutamate receptor *Grm5*, ionic glutamate receptor *Grin2c*, and thrombospondins *Thbs4* and *Thbs3* (Christopherson et al., 2005) were significantly altered (Supplementary Figure 3E), suggesting modulations in synaptic signaling during remyelination. Gene Set Enrichment Analysis (GSEA) further demonstrated a significant enrichment of genes functioning in cytokine signaling and phagocytosis during early demyelination (3weeks vs. Ctrl, Supplementary Figure 3G) and protein synthesis, amino acid metabolism, mitochondrial energy metabolism and reorganization of the extracellular matrix during the acute phase of remyelination (5 + 1weeks vs. ctrl, 5 + 1weeks vs. 3 weeks, Supplementary Figures 3F, H and Supplementary Table 3).

In comparison with acute phase, chronic de/remyelination was much less robust (Figures 3D, E). There was a lesser reduction of OL/myelin transcripts at 12 weeks of cuprizone over naïve controls than those at 3 weeks (Figure 3D). Moreover, when transcription factors/regulators involved in OL lineage cell development and differentiation (GO004879: OL differentiation, plus GO0140110: transcription regulator activation) were crossed to the DEG dataset of experimental samples, we found significant upregulation of *Sirt2* during remyelination of acutely demyelinated lesions but not chronic remyelination and only moderately upregulated at the late recovery time point (12 weeks after cuprizone cessation) (Figure 3E). In contrast, *Med12*, a component of Mediator Complex that regulates transcription initiation, was most significantly upregulated throughout the chronic phase (q < 3.53 × 10^–21^) but not in the acute phase (Figure 3E and Supplementary Table 1). *Med12* has been implicated in regulating terminal differentiation of OLs (Vogl et al., 2013), our data suggest that *Med12* may be critically involved in chronic de/remyelination. Interestingly, a cohort of transcription factors that are strongly expressed in OPCs and regulate OL lineage cell specification (*Ascl1/Mash1*) (Emery and Lu, 2015), proliferation (e.g., *Sox2*) (Zhang et al., 2018), and suppression of differentiation (e.g., *Hes5, Id2, Nkx6-2, Ctnb1*) (Emery and Lu, 2015), were distinctly downregulated in chronic de/remyelination (Figure 3E), suggesting OPC deficits in chronic lesions. Conversely, a cluster of genes critical to transcription-coupled DNA damage repair, *i.e., Ercc6, Xpc* and *Tardbp* (encoding TDP-43), was uniquely and significantly upregulated in the chronic demyelination paradigm (Figure 3E and Supplementary Table 2). These data suggest that defective DNA damage reparative mechanisms may underlie myelin repair aberrations following chronic demyelination. Importantly, these data reinforce the notion that acute and chronic remyelination may adopt differential molecular pathways, again underlying the importance of elucidating myelin regenerative mechanisms and functional consequences in the context of chronic demyelination.

Immune responses to demyelinating injury are integral to tissue recovery and myelin repair. Analysis of genes involved in inflammatory responses (gene ontology GO:0006954) uncovered gene clusters preferentially up- (group i) and down- (group ii) regulated in the chronic phase in comparison to acute de/remyelination (Figure 3F). Among those significantly upregulated genes in the chronic lesions are *Nfkbid* and *CD200r1*, negative regulators of immune responses. In contrast, many genes functioning in proinflammatory responses and leukocyte chemotaxis such as *Ly86, Cd14, Casp1*, *Ccl4, Cxcl10*, and *Ccl12* were downregulated in the chronic lesions (Figure 3F), consistent with immunohistochemical data showing that chronically demyelinating lesions exhibited blunted immune responses and micro/astrogliosis (Figures 1D, E). Further, eleven DEGs were found common to both the acute and chronic regenerative phases (Figure 3G). The expression levels of genes that were upregulated (*Gdf15, Trib3*, and *Cyb5r2)* and downregulated (e.g., *Gjc2* and *Abca8a*) during demyelination were essentially all restored over time during recovery (Figure 3G).

### Common and differential region-dependent regeneration responses following chronic demyelination

**FIGURE 4:**
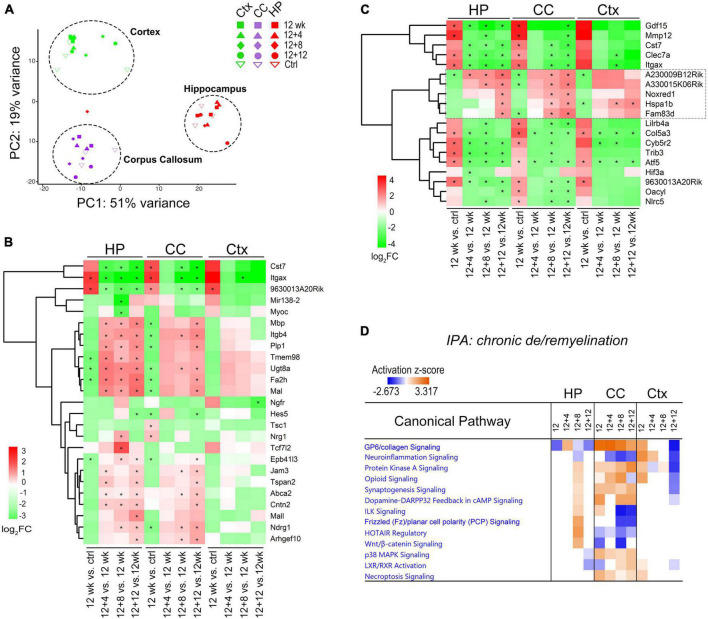
Comparative transcriptome analysis reveals brain-region dependent changes of canonical pathways during chronic de/remyelination. **(A)** Principal component analysis (PCA) of gene expression shows the separation of three sample groups corresponding to the brain regions: hippocampus (HP), corpus callosum (CC), and cortex (Ctx). Each dot represents an individual sample. **(B)** Relative expression changes of myelination related genes (GO0042552, 171 genes, *q* < 0.05) during recovery phase after chronic demyelination. A Heatmap of the top 25 highly modulated genes (ranked) is shown. Asterisks indicate *q* < 0.05. **(C)** Heatmap of 19 DEGs commonly modulated across the three brain regions (HP, CC, and Ctx). Asterisks indicate *q* < 0.05. **(D)** IPA comparison analysis across regions and time-points revealed specific canonical pathways activated in the corpus callosum. Positive (orange) and negative (blue) z-scores indicate predicted, respectively, activation and inhibition of specific pathways. Values of |z| > 2 are considered highly significant.

To determine whether chronic de/remyelination in different brain regions was transcriptionally distinct, we compared gene expression patterns of three brain regions, namely the hippocampus, corpus callosum, and cortex, after chronic cuprizone exposure and recovery time points up to 12 weeks (Figure 4A). Principal component analysis (PCA) of all experimental samples demonstrated overt group separation coinciding with the three brain regions (Figure 4A). We performed GSEA based on differential gene expression in the three brain regions between control and chronically demyelinated mice. GSEA demonstrated significant downregulation of pathways related to energy, amino acid, and lipid metabolism, protein membrane targeting and translation (Supplementary Figures 4A–C and Supplementary Table 4). While pathways related to stress responses, cell cycle, transcription, and mitosis were highly enriched in the hippocampus in comparison to controls, extracellular matrix (ECM) organization and collagen synthesis and signaling pathways were significantly enriched in corpus callosum and cortex (Supplementary Figures 4A–D). Interestingly, the chronically demyelinated corpus callosum exhibited highly enriched transcripts related to neuronal system and voltage-gated potassium channels including neuron-enriched *Kcnb2* (Boscia et al., 2021) (Supplementary Figure 4C and Supplementary Table 2).

We first examined myelin-related genes across these regions using myelination gene ontology GO:0042552 and compared relative changes during the remyelination phase (12 + 4, 12 + 8, 12 + 12 weeks) versus demyelination (12 weeks). Unbiased clustering of the top 25 ranked genes (log2 FC ≥ 1, q < 0.01) demonstrated relatively robust upregulation of myelin genes such as *Mbp, Plp1, Mag*, and *Mal* in the hippocampus and corpus callosum during the recovery, and less robust changes in the cortex (Figure 4B). Analysis of Venn diagrams of regional DEGs between recovery and demyelination showed significant DEGs common to all the three brain regions examined at either one or more recovering timepoints (Figure 4C). These include down-regulation of *Itgax* (CD11c), *Clec7a* (Dectin-1), and *Cst7*, which are associated with the subtype of disease-associated microglia. Most interestingly, we identified a group of overlapping genes during the remyelinating phase, including two highly modulated long non-coding (Lnc) RNAs (A230009B12Rik and A330015K06Rik) with currently unknown functions (4C, box) as well as *Noxred1* (NADP–Dependent Oxidoreductase Domain–Containing 1) and the heat shock gene *Hspa1b*, which was recently shown to be upregulated in oligodendrocytes from MS lesions as compared to controls (Jakel et al., 2019). Of note, the third LncRNA identified through our analysis was 9630013A20Rik (Figure 4C), a newly identified newborn immature OL-specific and chromatin-associated LncRNA (LncOL1, Pcdh17it) that regulates stage-specific OL differentiation (He et al., 2017; Kasuga et al., 2019).

**FIGURE 5:**
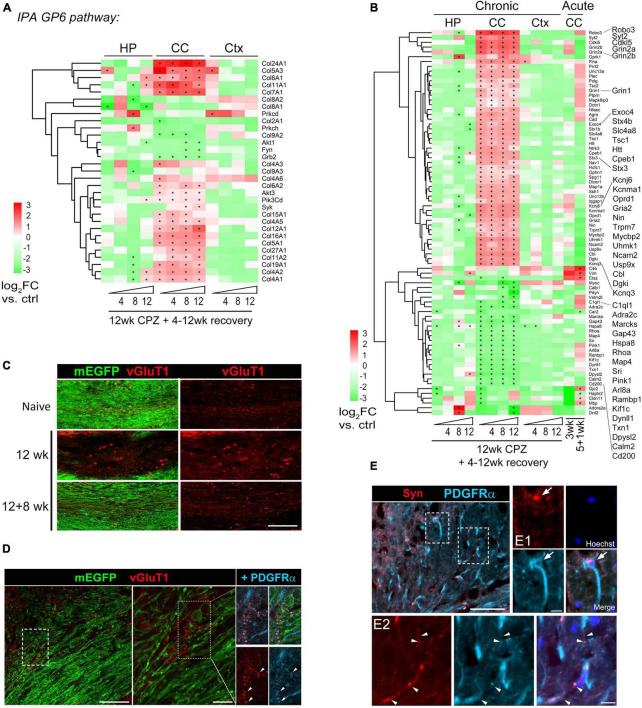
Activation of GP6/collagen and synaptogenesis pathways in corpus callosum during chronic demyelination and recovery. **(A)** Heatmap of 31 DEGs functioning in the GP6 pathway identified by IPA as the most significantly altered pathway in the corpus callosum. Region- and time-point comparison of the 31 DEGs over normal controls is shown. Asterisks indicate significant DEGs (*q* < 0.05) in comparison to controls. **(B)** Axon gene ontology (GO0030424: 765 genes) was applied to the Deseq dataset and Log2FC Heatmap of top 80 DEGs during acute and chronic demyelination and remyelination is shown. Asterisks, significant DEGs in comparison to controls (q < 0.05). **(C)** Representative vGluT1 staining of the medial corpus callosum of control mice and mice treated with cuprizone for 12 weeks followed by 8 weeks of recovery. Scale bar, 100 μm. **(D)** Representative confocal image of lateral corpus callosum from CNP-mEGFP mice fed cuprizone for 12 weeks. The boxed regions are zoomed-in on the right. Scale bars, 50 μm (left), 10 μm (right). **(E)** Representative synaptophysin (Syn) and PDGFRα double immunostaining images of corpus callosum sections from 12 wk cuprizone mice. Scale bar, 50 μm. Boxed regions are shown in higher magnification in panels (E1,E2). Arrows indicate a Syn^+^ OPC and arrowheads indicate overlapping synaptophysin and PDGFRα immunoreactivity. Scale bars, 10 μm (E1,E2).

Ingenuity Pathway Analysis (IPA) was used to evaluate molecular pathway changes during chronic de/remyelination across the three different brain areas (Figure 4D). We detected glycoprotein VI (GP6), protein kinase A (PKA) signaling and synaptogenesis as top signaling pathways activated in the corpus callosum. In contrast, neuroinflammation signaling was downregulated during chronic recovery and as compared to acute de/remyelination (Figure 4D and Supplementary Figure 4E). Interestingly, while the GP6 signaling pathway gradually decreased as remyelination proceeded, the PKA pathway gradually increased during chronic remyelination (Figure 4D), in support of the notion that PKA activation and CREB phosphorylation enhance oligodendrocyte maturation likely via neuronal/axonal signals (Malone et al., 2013; Qin et al., 2017). As GP6 is not expressed in the brain, we found that under the GP6 pathway defined by IPA, diverse subtypes of collagen genes were in fact highly upregulated in the corpus callosum, most notably *Col24A1, Col5A3, Col6A1, Col11A1, Col7A1, Col12A1* and *Col16A1* (Figure 5A). GSEA results further strengthened IPA analyses as collagen formation and ECM organization were consistently the top pathways enriched in the chronically demyelinated corpus callosum (Supplementary Figure 4). ECM gene ontology analysis (GO:0031012) of the corpus callosum samples from acute and chronic de/remyelination showed that the majority of collagens were significantly upregulated only in the chronic phase (Supplementary Figure 5A). In addition, thrombospondin 3 (*Thbs3*), tenascin-R (*Tnr*), and bone morphogenetic protein 1 (*Bmp1*) were also markedly upregulated in chronic de/remyelination. In contrast, transcripts of other ECM proteins such as laminins, tenascin, fibulin, fibronectin, and fibrinogen were not significantly different between chronic de/remyelination and normal controls. Together, these results suggest that ECM reorganization/signaling is a prominent feature of the white matter in chronic versus acute demyelination. The data also demonstrate that myelin recovery in the chronically demyelinated white matter can occur successfully in the rodent brain despite of the persistent upregulation of collagen-related ECM pathways.

As the collagen/ECM pathway was the top canonical pathway identified in the chronically de/remyelinating white matter in mice, we asked if this is relevant to human MS white matter lesions. We analyzed DEGs of different lesion types in the white matter of patients with progressive MS in comparison to controls (Elkjaer et al., 2019) and indeed found significant aberrations of the collagen/ECM related pathways in progressive MS (Supplementary Figure 5 and Supplementary Table 5). Of note, while some transcripts (e.g., *COL24A1, COL5A1/A3, COL6A1*) were significantly upregulated in both chronic cuprizone mice and specific MS lesion types, others had opposite trend (e.g., *COL16A1*) or only highly upregulated in the MS white matter but not in mice subjected to chronic cuprizone exposure (e.g., *COL1A1/A2, COL8A1/A2*).

### Chronic de/remyelination of the white matter tract is associated with activation of axon/synaptogenesis signaling pathway

Cross region pathway analysis unexpectedly revealed activation of a synaptogenesis signaling pathway in the corpus callosum but not in the cortex and hippocampus during the chronic phase of de/remyelination (Figure 4D). We examined each gene further using the axon Gene Ontology term (GO:0030424) and identified top 80 differentially modulated genes (Figure 5B and Supplementary Table 5). Of note, glutamatergic signaling was selectively upregulated in the corpus callosum throughout all chronic timepoints (Figure 5B). In particular, glutamate-gated NMDA receptors (*Grin2a, Grin2b*) and AMPA receptor *Gria2* were significantly upregulated in chronically demyelinating and remyelinating corpus callosum. Genes functioning in axonal navigation (*Robo3*), synaptic vesicles (*Sty2, Stx1b, Stx4b, Unc13, Exoc4*), and potassium channels (*Kcnq3, Kcnj6, Kcnma1*) were also upregulated selectively in the corpus callosum in comparison to gray-matter region genes changes (Figure 5B). Overall, the expression profile of axon-related DEGs demonstrated that chronic de/remyelination of the callosal white matter was associated with persistent activation of axon/synaptogenesis signaling pathway. This long-lasting derivation of genes related to axonal/synaptic functions, even after 12 weeks of recovery when most of callosal axons were remyelinated, may underlie the behavioral aberrations developed over time after the chronic insult (Figures 2H, I). Moreover, axon GO analysis of the DEG dataset of MS white matter lesions (Elkjaer et al., 2019) showed significant upregulation of transcripts involved in potassium homeostasis and glutamate receptor signaling (e.g., *KCNJ6, KCNMA1, GRIN2B*), particularly in chronic active MS lesions (Supplementary Figure 5C and Supplementary Table 5).

Given that glutamate receptors and synaptic vesicle pathways were found persistently upregulated in the corpus callosum during chronic de/remyelination and that neuronal glutamatergic signaling stimulates OPC differentiation (Kukley et al., 2007; Ziskin et al., 2007; Wake et al., 2011; Hines et al., 2015; Mensch et al., 2015), we next examined vesicular glutamate transporter protein vGluT1 in the corpus callosum by immunohistochemistry. Chronic cuprizone treatment resulted in the significant formation of vGluT1^+^ spheroids in the white matter, suggesting axonal damage and blockade of axonal trafficking (Figure 5C). Interestingly, whereas the vast majority of vGluT1 immunoreactivity did not overlap with mEGFP^+^ myelin sheaths or plasma membranes of mature oligodendrocytes, punctate vGluT1^+^ immunosignal overlapped frequently with OPC marker PDGFRα in remyelinating areas (Figure 5D). This observation is consistent with the notion that activity-dependent axonal vesicular glutamate release activates OPCs and regulates myelination (Kukley et al., 2007; Ziskin et al., 2007; Mensch et al., 2015; Wake et al., 2015) and remyelination (Gautier et al., 2015). Moreover, chronic de/remyelination also induced accumulations of synaptophysin, an integral membrane protein of presynaptic vesicles, in the white matter tract (Figure 5E). Many synaptophysin^+^ puncta were found overlapping with PDGFRα^+^ OPC processes (Figures 5E1, E2), reinforcing axonal presynaptic inputs onto OPCs in the context of chronic de/remyelination. Together, the data suggest that glutamatergic signaling between unmyelinated callosal axons and OPCs may underline wide-spread remyelination of chronically demyelinated axons. However, excessive and prolonged activation of the glutamatergic pathway may also exacerbate excitotoxic damage in chronic de/remyelinating lesions.

## Discussion

In this study we investigated spatiotemporal transcriptome changes, myelin repair, and functional recovery following chronic demyelinating insult. Using the highly trackable cuprizone model of CNS demyelination, we demonstrate significant spontaneous remyelination in both white matter and gray matter over an extended recovery period after chronic demyelination. Gliosis, neuroinflammation and remyelination during the chronic phase were much less pronounced than those following acute demyelination. Axonal damage was evident at ultrastructural levels in the chronically demyelinated corpus callosum. Remyelinated SMI32^+^ axons were frequently observed in the cortex during the chronic recovery phase, suggesting prolonged local axonopathy. Unexpectedly, we found that despite wide-spread myelin return, the mice progressively developed new bilateral sensorimotor coordination deficits months after the cessation of chronic cuprizone treatment. Unbiased tissue transcriptome analysis revealed region- and time-dependent gene alterations in the chronic de/remyelination paradigm and further underscored differences of reparative processes in chronic and acute lesions. We identified that the GP6/collagen pathway was markedly upregulated in chronically de/remyelinating white matter as compared to other brain regions or to acutely de/remyelinated lesions, suggesting a previously unrecognized function of collagen signaling in remyelination. Consistent with the concept of neuronal activity-dependent regulation of myelination (Gibson et al., 2014; McKenzie et al., 2014; Hines et al., 2015; Hughes et al., 2018; Ortiz et al., 2019), we found that chronic de/remyelination is associated with persistent upregulation of axon-relevant transcripts in the white matter, particularly those involved in glutamatergic synaptogenesis. Consistently, we found that vGluT1^+^ and presynaptic vesicles in the remyelinating white matter often overlap with OPC processes, suggesting OPC-axon synaptic interactions. Together, these data suggest a potential mechanistic link between elevated excitatory axonal activity and myelin repair during chronic de/remyelination, but at the same time also raise the possibility of prolonged overactivation of axons leading to increased neuronal vulnerability.

Remyelination is a spontaneous regenerative process that occurs following demyelination but often fails in demyelinating diseases such as MS (Franklin and Ffrench-Constant, 2008). Efficient myelin return is critical in preventing irreversible axonal loss and promoting functional recovery. Remyelination restores trophic and metabolic support to the axon and reestablishes saltatory conduction via the reorganization of Na^+^ channels at the nodes (Trapp and Stys, 2009). Regenerated myelin sheaths, although thinner and shorter than original sheaths, are associated with recovery of function (Franklin and Ffrench-Constant, 2008). The mechanisms of remyelination have been investigated extensively following acute demyelination, yet myelin repair following a chronic demyelinating insult is less studied, even though chronic de/remyelination may exhibit more relevance with MS demyelinating lesions. To better understand CNS myelin repair in the context of chronic de/remyelination, we performed RNA-seq of the corpus callosum, cortex, and hippocampus during the chronic phase of demyelination and at various recovery timepoints after the cessation of chronic cuprizone exposure (Figure 4). Furthermore, we compared transcriptome profiles of the corpus callosum following acute and chronic demyelination and remyelination (Figure 3). We found shared and distinct characteristics of myelin repair temporally as well as regional differences in the chronic phase. Overall, glial responses, inflammation, and OL/myelin regeneration were robust and rapid in the acute phase (Figures 3D, F). In contrast, OL/myelin regeneration in the chronic phase followed a much slower trajectory with subdued inflammatory responses and was burdened with increased DNA damage stress (Figures 3D–F). At the transcriptome level, the three brain regions examined displayed variable extents of downregulation of OL/myelin-related genes during chronic de/remyelination with corpus callosum being the most robust, followed by hippocampus and the least alterations in the cortex (Figure 4 and Supplementary Table 2). The subtler differential OL/myelin transcriptome changes in the cortex are likely due to the fact that these were bulk RNA-seq analyses of entire neocortices and/or that chronic demyelination is accompanied by ongoing remyelination. This is in agreement with the observation that cortical remyelination occurs more extensively than the white matter in patients with chronic MS (Albert et al., 2007). At protein levels, cortical de/remyelination were clearly evident (Figures 1, 2). Using a transgenic mouse line where membrane-tethered fluorescent EGFP is spontaneously expressed in mature OLs and myelin sheaths, thereby providing sensitive and direct visualization of OL membranes (Deng et al., 2014), we observed many myelinating OLs in cortices 8 weeks after chronic cuprizone treatment (Figure 2E). Interestingly, multiple damaged axon segments, as indicated by SMI-32 immunoreactivity, were myelinated at this recovery time point. Remyelination of acutely damaged axons has also been observed in MS lesions (Schultz et al., 2017). In our study, it is not possible to discern whether these mEGFP^+^ SMI-32^+^ axons are damaged axon regions that were subsequently remyelinated or whether these are remyelinated axons that continued to degenerate, the latter of which would have important implications for the progressive stage of MS, where progressive and irreversible loss of axons and neuronal atrophy accounts for the progressive nature of the disease. In the current study, we have not examined focal axonal damage in the cortices of mEGFP transgenic mice beyond the 8 weeks of recovery time point. Additionally, ongoing axonal damage months after demyelination could be independent of remyelination. As such, it would be important in future studies to investigate mechanisms underlying cortical axonal degeneration in chronic de/remyelination settings and develop mechanism-based intervention and neuroprotective strategies.

Our pathway analysis of differentially expressed genes across times and brain regions identified multiple region-specific as well as shared canonical pathways associated with chronic de/remyelination. These datasets shall provide a road map for further interrogating cellular and molecular events that contribute to myelin regeneration and modulation of axonal functions. The GP6/collagen signaling pathway, a top upregulated pathway found in the white matter under the chronic paradigm, is somewhat unexpected (Figures 4, 5). Although ECM proteins and modifiers such as laminin, hyaluronan, and fibronectin have been implicated in modulating tissue regeneration including remyelination (Ghorbani and Yong, 2021), collagens are in general minimally expressed in the healthy brain. At present, little is known about the role of collagens in the context of remyelination. Increased type IV collagen in MS lesions is thought to inhibit OPC migration (van Horssen et al., 2006). Of the collagens persistently upregulated in chronic de/remyelinated corpus callosum (Figure 5A), many are constitutively expressed at low levels in murine CNS cells in development (Zhang et al., 2014). In progressive MS white matter lesions, multiple collagen transcripts are upregulated with some unique alterations not found in the chronically de/remyelinated white matter in mice (Supplementary Figure 5B). Thus, upregulation of the GP6/collagen pathway in chronic de/remyelinating white matter suggests a previously unrecognized role for collagens in the context of chronic demyelination and may have relevance to progressive MS and remyelination failure.

Another interesting finding from our pathway analysis was the prolonged upregulation of axon-related transcripts involved in synaptogenesis, particularly those related to glutamatergic signaling in chronic white matter lesions (Figure 5B). It is generally acknowledged in the field that oligodendrocyte lineage cells express functional glutamate receptors including NMDA receptors and AMPA/kainate receptors and that OPCs receive neuronal synaptic input, one of the mechanisms by which neuronal activity modulates developmental and adaptive myelination (Bergles and Richardson, 2015). Moreover, increased neuronal activity has been recently shown to enhance functional myelin repair (Ortiz et al., 2019; Bacmeister et al., 2020; Orthmann-Murphy et al., 2020). Demyelinated axons may be more electrically active and produce more synaptic vesicles to increase OPC differentiation, promote remyelination and stabilize nascent myelin sheaths (Hines et al., 2015; Ortiz et al., 2019; Pfeiffer et al., 2019). Further, remyelination may be regulated via neuronal activity specifically in the form of glutamatergic signaling to OPCs (Gautier et al., 2015). Our transcriptome analysis and double immunohistochemistry showed that presynaptic vesicles, in particular vGluT1^+^ synaptic vesicles, co-localized to OPC processes, providing further support to the concept that demyelinated axons are more electrically active in an attempt to promote myelin return and compensate the loss of myelin. On the other hand, as callosal axons arise primarily from layers II/III and V pyramidal neurons that are excitatory, the findings of persistent upregulation of glutamatergic signaling, even at 12 weeks after the removal of cuprizone, also suggest a mechanism by which chronic, ongoing axonal degeneration may originate from excitotoxic damage due to prolonged chronic over-activation.

The functional outcome of cuprizone-induced CNS de/remyelination, particularly motor functional impairments, has been examined in multiple studies (Lubrich et al., 2022). However, it is still unclear to what extent functional impairments recover upon remyelination, particularly in the context of chronic de/remyelination. In this study we used the sensitive DigiGait motorized treadmill instead of the widely employed rotarod test and uncovered new motor function deficits that gradually developed after the withdrawal of the chronic demyelinating insult (Figures 2H, I). This recovery phase-dependent evolvement of functional deficits is unexpected, but in line with a previous study that found declined complex wheel-running performance 6 months after remyelination in a recurrent acute cuprizone model (Manrique-Hoyos et al., 2012). In that study, locomotor performance decline was associated with loss of callosal axons, consistent with TEM observations of degenerating callosal axons in the 12 + 12 weeks mice. Thus, accumulation of low level ongoing axonal damage likely underlies the deterioration of motor functions over time. Another possibility, though not mutually exclusive, is that the pattern of myelination along denuded axon segments changes over time during chronic de/remyelination as cortical axons are adept to more dynamic myelination (Orthmann-Murphy et al., 2020), and as such the patterns of regenerated myelin may re-adjust motor neural circuitry and contribute to functional alterations.

In summary, we showed that myelin repair processes in response to chronic demyelination exhibit unique characteristics from those of acute demyelination. We identified multiple genes and specific pathways associated with chronic demyelination and remyelination. We further found prolonged local axonal damage in the cortical gray matter and the evolvement of altered motor functions upon extended periods of recovery. Our study underscores the importance of developing axonal protection strategies in addition to better myelin repair and has implications for progressive MS.

## Data availability statement

The RNA-seq datasets are deposited in the Gene Expression Omnibus data repository, accession number: GSE212147. Data presented in this study can be found in the article/Supplementary material.

## Ethics statement

This animal study was reviewed and approved by IACUC at the Texas A&M University.

## Author contributions

GS, SK, and JL designed the experiments, analyzed data, and wrote the manuscript. GS performed most of the experiments. SK conducted acute cuprizone experiments and performed IPA and GSEA. DM performed intoxication of mEGFP transgenic mice. SK and GS did tissue dissections and RNA preparations. AH prepared cDNA libraries and carried out some of the RNA-seq experiments. KK, SK, and JL analyzed RNA-seq data. JS assisted with electron microscopy. JL analyzed TEM data. All authors contributed to the article and approved the submitted version.

## Footnotes

1
http://bioinformatics.psb.ugent.be/webtools/Venn/

## Supplementary material

Supplementary Figure 1 Acute and chronic cuprizone exposure induces cortical demyelination followed by remyelination. **(A,B)** Representative images of the somatosensory cortex directly visualized with CNP-mEGFP expression and immunostained for Iba1. Scale bar, 200 μm.

Supplementary Figure 2 DigiGait and open field behavioral analyses of age-matched control and cuprizone mice at various time points following chronic demyelination. **(A)** DigiGait behavioral metrics of mice after chronic de/remyelination and of age-matched control mice. Data are mean ± SD of averaged stride frequency and stride length of hindpaws of individual animals, averaged stance width and absolute paw angle of forepaws of each animal, and the average gait symmetry between all four paws of each animal (gait symmetry). **(B)** Open Field behavioral analysis of mice after chronic de/remyelination and of age-matched control mice. Data are mean ± SD of the distance traveled around the chamber, move time, jumps, margin time, and center time over the experimental period of 30 min. Data points represent individual animals (*n* = 8–11 mice per time point). **p* < 0.05; ^**^*p* < 0.01; ^***^*p* < 0.001; ^****^*p* < 0.0001. One-way ANOVA with Tukey’s *post hoc* correction.

Supplementary Figure 3 Cell type-annotation of DEGs between acute demyelination and remyelination samples and top altered cellular pathways identified by Gene Set Enhancement Analysis. **(A)** Schematic of experiments for acute cuprizone intoxication and tissue analysis. **(B)** Heatmap of 52 OL-enriched genes significantly (Log2FC > | 2|, q < 0.05) modulated during the acute phase of remyelination (5 + 1 week) versus demyelination (3 weeks). Heatmap shows gene expression values for the 52 OL genes from 3 mice at specified time points. Genes are hierarchically clustered based on correlation with expression across each sample. **(C–E)** Volcano plots showing the log2 fold change and the significance of DEGs of oligodendrocytes-, microglia/macrophages-, and astrocytes-enriched genes between 5 + 1 weeks over 3 weeks samples. Genes highlighted in red: OL, Log2FC > | 1.5|, *p* < 5 × 10^–5^; microglia and astrocytes, Log2FC > | 1.0|, *p* < 5 × 10^–5^. **(F–H)** Gene Set Enrichment Analysis (GSEA) identified significantly enriched pathways that were up- or downregulated during acute demyelination (3 weeks vs. control) and early remyelination (5 + 1 weeks vs. 3 weeks, and 5 + 1 weeks vs. control). Top pathways with normalized enrichment score (NES) great than | 2.5| and False Discovery Rate (FDR) *q* value at least less than 10^–3^ were shown.

Supplementary Figure 4 Top enriched pathways up- and down-regulated in the hippocampus, corpus callosum, and cortex after chronic demyelination. **(A–D)** GSEA identified highly enriched pathways altered during chronic demyelination (12 weeks vs. control and 12 + 4 weeks vs. 12 weeks). Gene sets were ordered by normalized enrichment score (NES) and top 10 pathways with FDR q value of 0, and at least less than 1 × 10^–3^ were displayed. **(E)** IPA canonical pathway comparison of corpus callosum samples following acute and chronic de/remyelination.

Supplementary Figure 5 ECM and axon related gene changes in the murine white matter following acute and chronic de/remyelination and in MS white matter lesions from postmortem brain tissues of human patients with progressive multiple sclerosis. **(A)** ECM gene ontology (GO:0031012: total 531 genes) was applied to the Deseq dataset and a Heatmap of 116 DEGs (Log2FC > | 1|, q < 0.05) of the corpus callosum during acute and chronic de/remyelination is shown. Asterisks, significant DEGs when compared to controls (q < 0.05). **(B**,**C)** ECM GO:0031012 and Axon GO:0030424 were applied to the human MS DEGs dataset generated from transcriptome analysis of 4 types of progressive MS white matter lesions in comparison to control white matter (GSE138614) (Elkjaer et al., 2019). Progressive MS (PMS) white matter (WM) lesions were classified as active lesion (AL), chronic active lesion (CA), inactive lesion (IL) and repair/early remyelinating lesion (RL) based on MOG^+^ and HLA-DR^+^ immunostaining patterns. Heatmaps were generated using a cut off of Log2FC > | 1| and FDR < 0.05 and DEGs with NA values removed. For Axon GO heatmap, only upregulated DEGs are shown. See Supplementary Data Table 5 for DEGs of the four types of human progressive MS white matter lesions versus control white matter.
