# Supplementary figures and images for: Brain region dependent molecular signatures and myelin repair following chronic demyelination
Source: Front Cell Neurosci. 2023 Apr 26;17:1169786. doi: 10.3389/fncel.2023.1169786 (PMC10171432; doi:10.3389/fncel.2023.1169786)

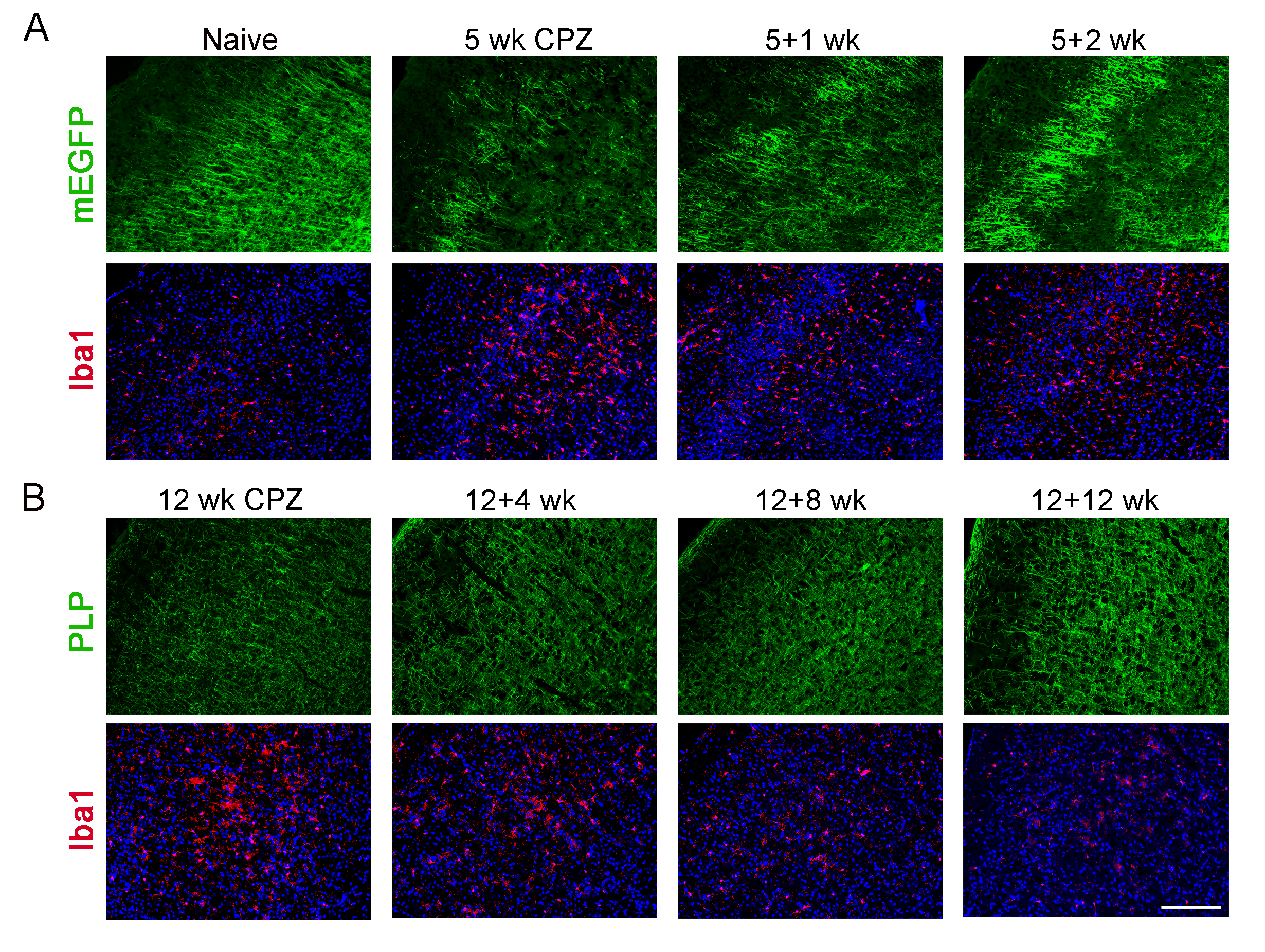

Supplement: Supplementary Figure 1 — Acute and chronic cuprizone exposure induces cortical demyelination followed by remyelination. (A,B) Representative images of the somatosensory cortex directly visualized with CNP-mEGFP expression and immunostained for Iba1. Scale bar, 200 μm. [file Image_1.jpeg]

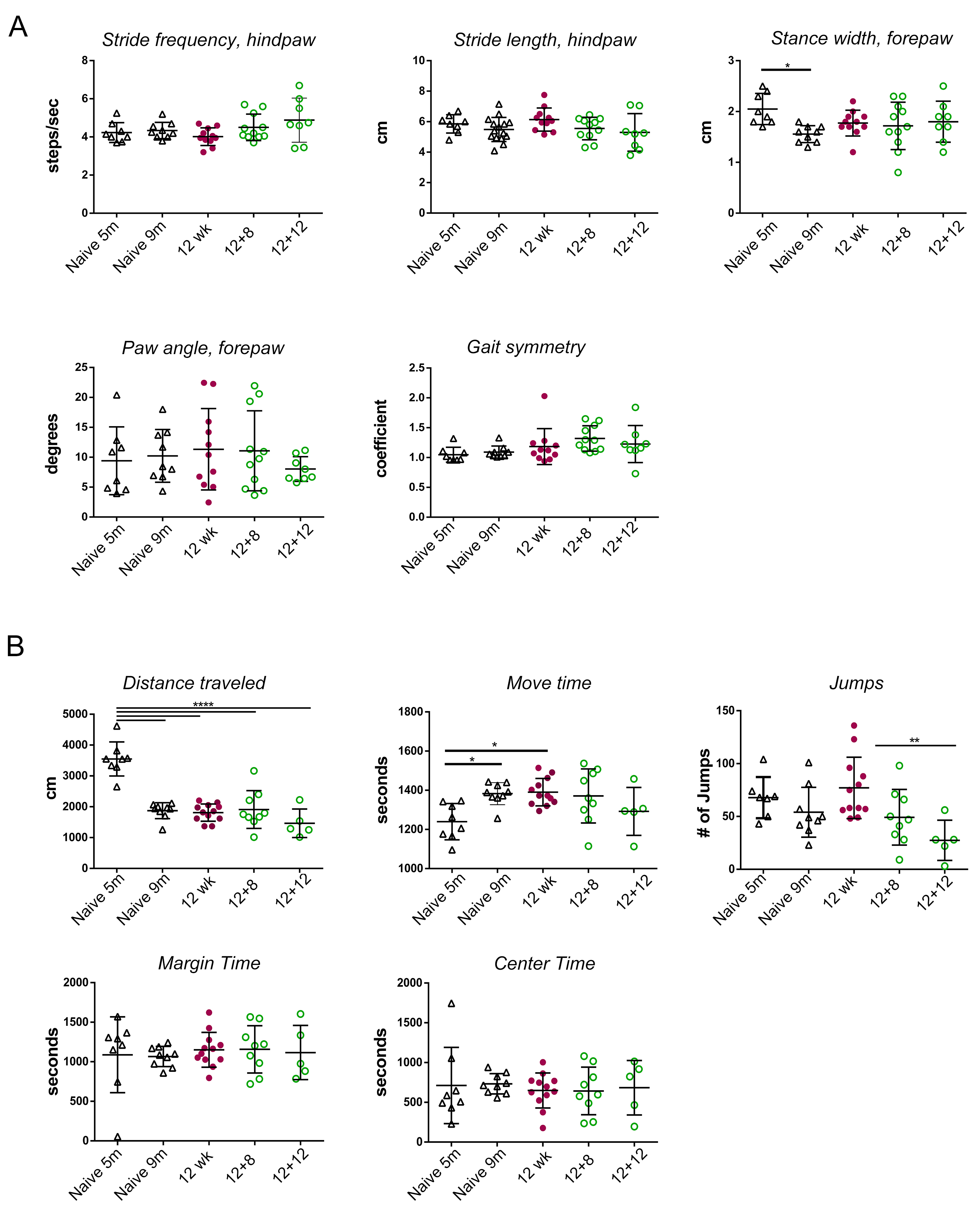

Supplement: Supplementary Figure 2 — DigiGait and open field behavioral analyses of age-matched control and cuprizone mice at various time points following chronic demyelination. (A) DigiGait behavioral metrics of mice after chronic de/remyelination and of age-matched control mice. Data are mean ± SD of averaged stride frequency and stride length of hindpaws of individual animals, averaged stance width and absolute paw angle of forepaws of each animal, and the average gait symmetry between all four paws of each animal (gait symmetry). (B) Open Field behavioral analysis of mice after chronic de/remyelination and of age-matched control mice. Data are mean ± SD of the distance traveled around the chamber, move time, jumps, margin time, and center time over the experimental period of 30 min. Data points represent individual animals (n = 8–11 mice per time point). *p < 0.05; **p < 0.01; ***p < 0.001; ****p < 0.0001. One-way ANOVA with Tukey’s post hoc correction. [file Image_2.jpeg]

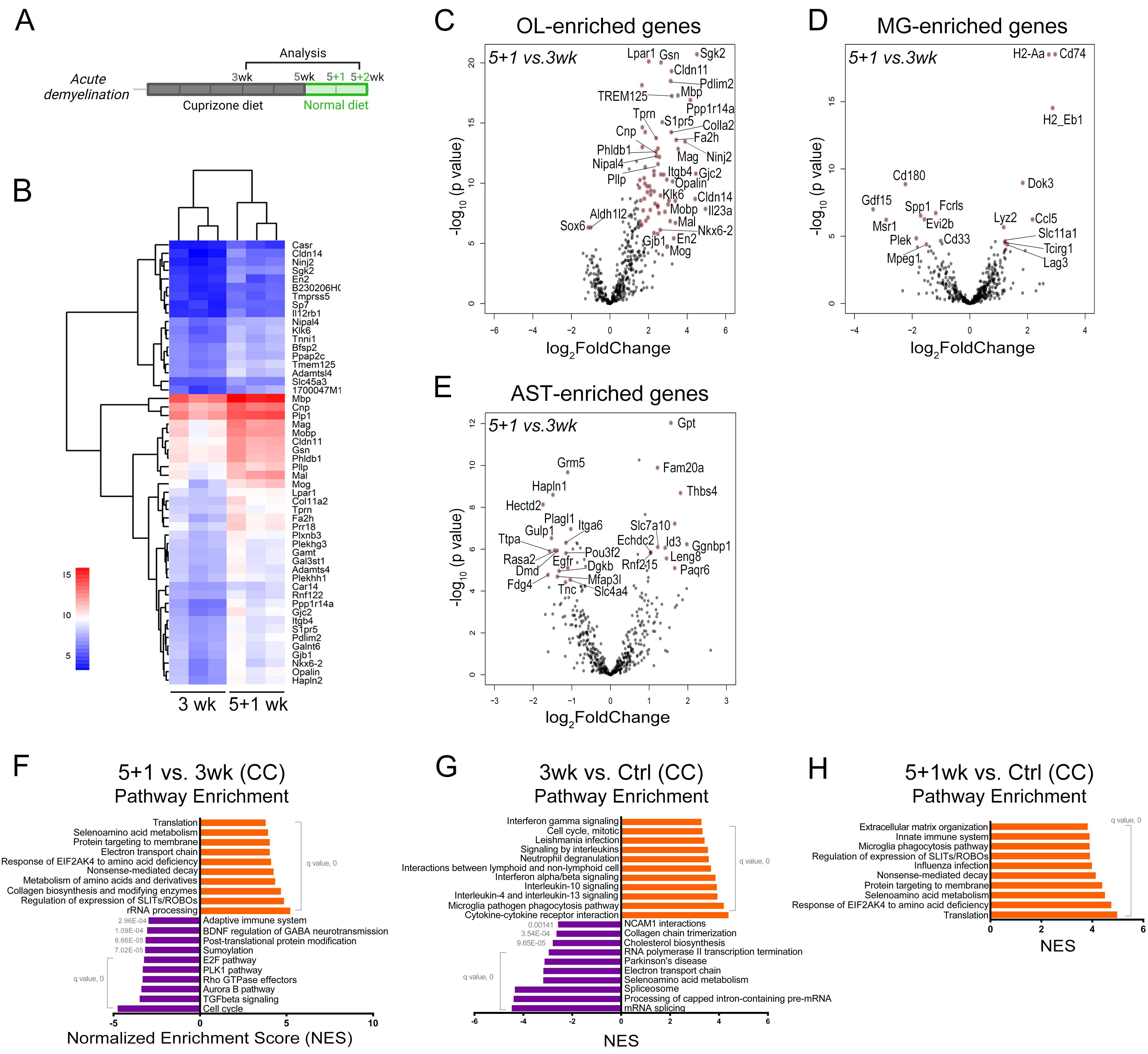

Supplement: Supplementary Figure 3 — Cell type-annotation of DEGs between acute demyelination and remyelination samples and top altered cellular pathways identified by Gene Set Enhancement Analysis. (A) Schematic of experiments for acute cuprizone intoxication and tissue analysis. (B) Heatmap of 52 OL-enriched genes significantly (Log2FC > | 2|, q < 0.05) modulated during the acute phase of remyelination (5 + 1 week) versus demyelination (3 weeks). Heatmap shows gene expression values for the 52 OL genes from 3 mice at specified time points. Genes are hierarchically clustered based on correlation with expression across each sample. (C–E) Volcano plots showing the log2 fold change and the significance of DEGs of oligodendrocytes-, microglia/macrophages-, and astrocytes-enriched genes between 5 + 1 weeks over 3 weeks samples. Genes highlighted in red: OL, Log2FC > | 1.5|, p < 5 × 10^–5^; microglia and astrocytes, Log2FC > | 1.0|, p < 5 × 10^–5^. (F–H) Gene Set Enrichment Analysis (GSEA) identified significantly enriched pathways that were up- or downregulated during acute demyelination (3 weeks vs. control) and early remyelination (5 + 1 weeks vs. 3 weeks, and 5 + 1 weeks vs. control). Top pathways with normalized enrichment score (NES) great than | 2.5| and False Discovery Rate (FDR) q value at least less than 10–3 were shown. [file Image_3.jpeg]

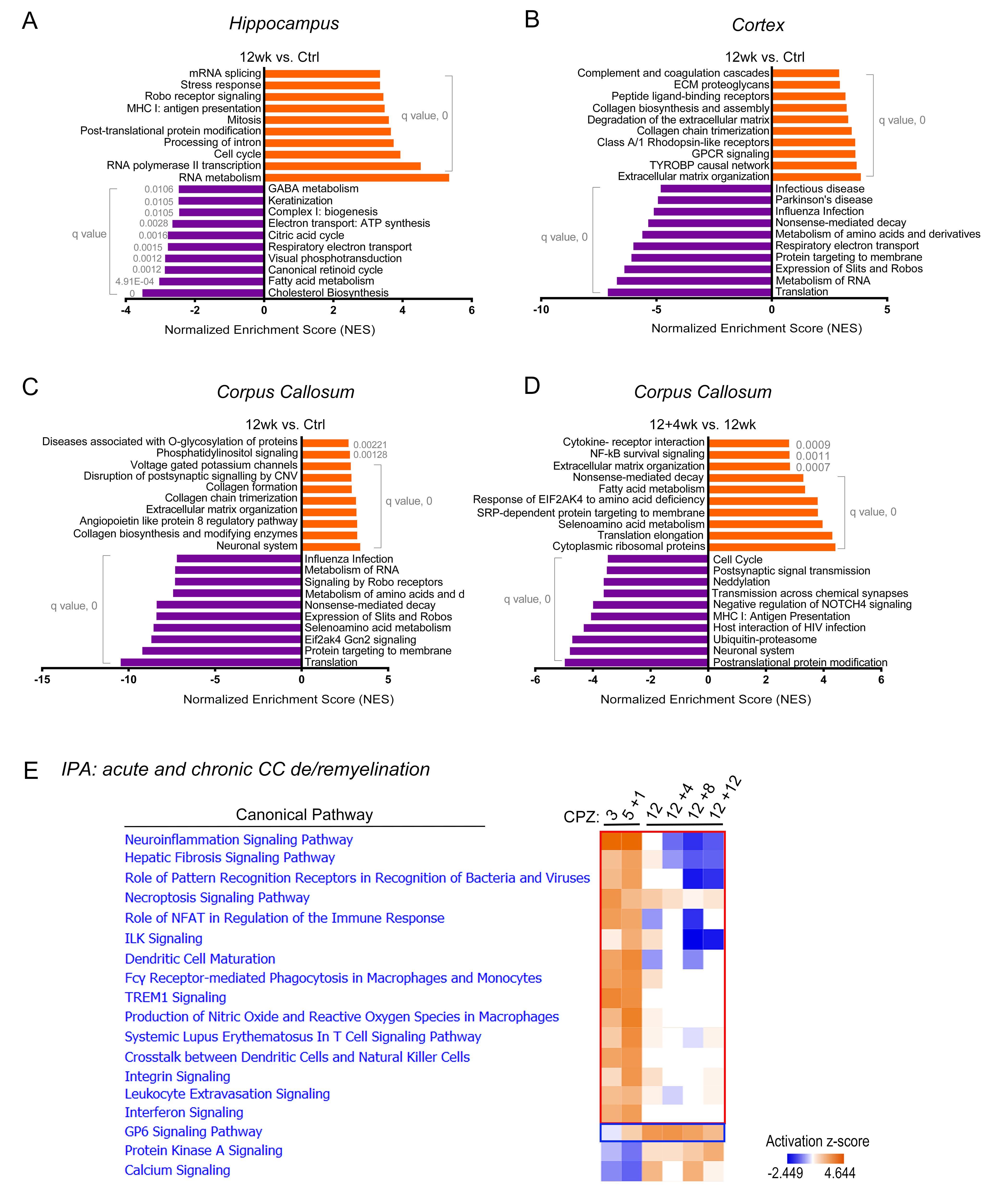

Supplement: Supplementary Figure 4 — Top enriched pathways up- and down-regulated in the hippocampus, corpus callosum, and cortex after chronic demyelination. (A–D) GSEA identified highly enriched pathways altered during chronic demyelination (12 weeks vs. control and 12 + 4 weeks vs. 12 weeks). Gene sets were ordered by normalized enrichment score (NES) and top 10 pathways with FDR q value of 0, and at least less than 1 × 10^–3^ were displayed. (E) IPA canonical pathway comparison of corpus callosum samples following acute and chronic de/remyelination. [file Image_4.jpeg]

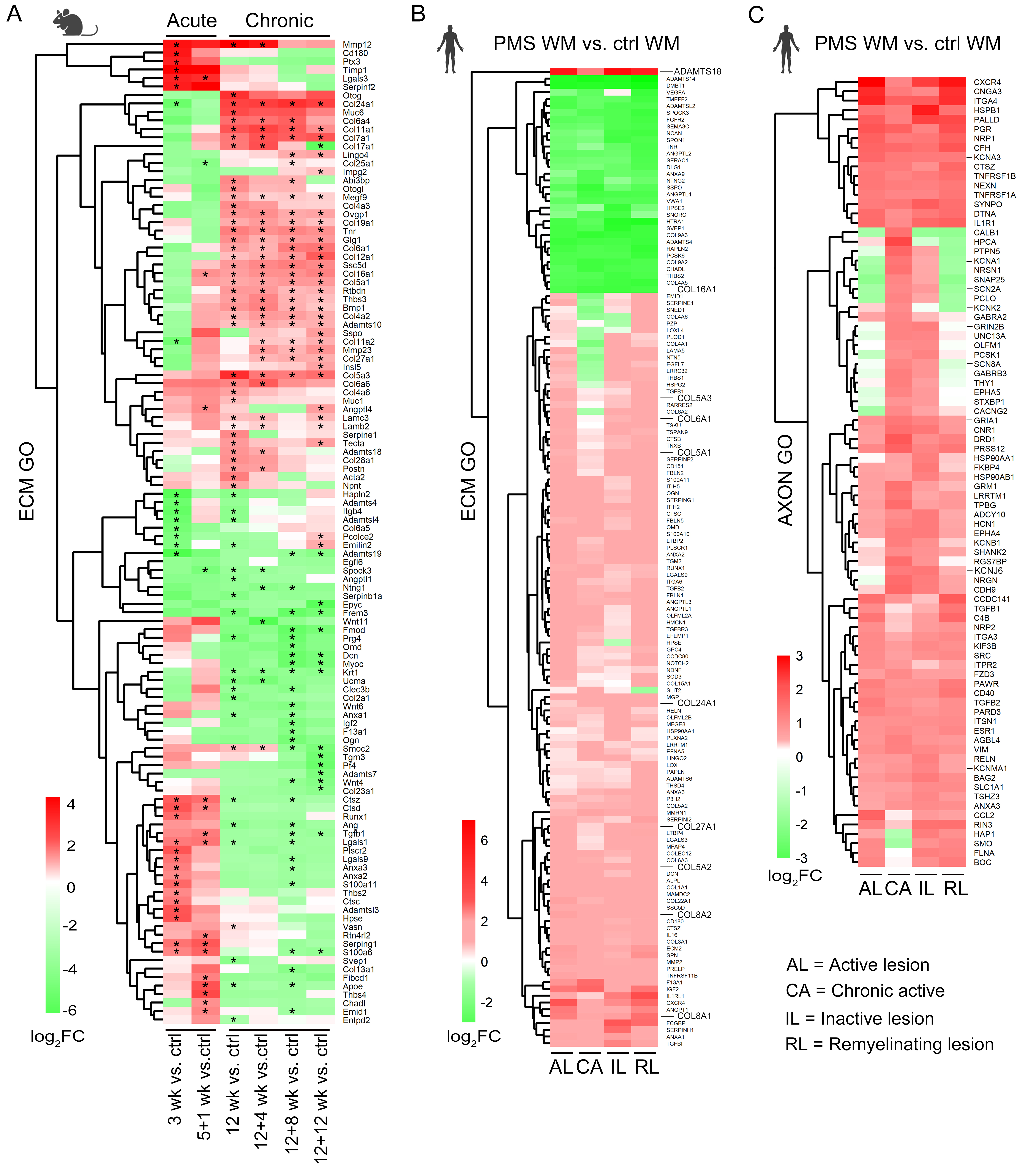

Supplement: Supplementary Figure 5 — ECM and axon related gene changes in the murine white matter following acute and chronic de/remyelination and in MS white matter lesions from postmortem brain tissues of human patients with progressive multiple sclerosis. (A) ECM gene ontology (GO:0031012: total 531 genes) was applied to the Deseq dataset and a Heatmap of 116 DEGs (Log2FC > | 1|, q < 0.05) of the corpus callosum during acute and chronic de/remyelination is shown. Asterisks, significant DEGs when compared to controls (q < 0.05). (B,C) ECM GO:0031012 and Axon GO:0030424 were applied to the human MS DEGs dataset generated from transcriptome analysis of 4 types of progressive MS white matter lesions in comparison to control white matter (GSE138614) (Elkjaer et al., 2019). Progressive MS (PMS) white matter (WM) lesions were classified as active lesion (AL), chronic active lesion (CA), inactive lesion (IL) and repair/early remyelinating lesion (RL) based on MOG+ and HLA-DR+ immunostaining patterns. Heatmaps were generated using a cut off of Log2FC > | 1| and FDR < 0.05 and DEGs with NA values removed. For Axon GO heatmap, only upregulated DEGs are shown. See Supplementary Data Table 5 for DEGs of the four types of human progressive MS white matter lesions versus control white matter. [file Image_5.jpeg]
